# Seroprevalence of contagious ecthyma in goats of Assam: An analysis by indirect enzyme-linked immunosorbent assay

**DOI:** 10.14202/vetworld.2016.1028-1033

**Published:** 2016-09-28

**Authors:** Mousumi Bora, Durlav Prasad Bora, Nagendra Nath Barman, Biswajyoti Borah, Sutopa Das

**Affiliations:** 1 Division of Virology, Indian Veterinary Research Institute, Izatnagar, Bareilly, Uttar Pradesh, India; 2 Department of Microbiology, College of Veterinary Science, Assam Agricultural University, Khanapara Campus, Guwahati, Assam, India; 3 Department of Animal Biotechnology, Assam Agricultural University, Khanapara Campus, Guwahati, Assam, India

**Keywords:** Assam, contagious ecthyma, goats, indirect enzyme linked immuno sorbent assay, serosurveillance

## Abstract

**Aim:**

The objective of this study was to screen the prevalence of contagious ecthyma (CE) among the goat population of Assam owing to its high prevalence rate.

**Materials and Methods:**

In this study, a total of 231 serum samples were collected from 12 districts of Assam during September 2013 to July 2014. The serum samples were tested for the presence of antibodies against Orf virus (ORFV) by indirect enzyme-linked immunosorbent assay (ELISA). Indirect ELISA was standardized using purified Orf reference virus produced in bulk in primary lamb testes cells.

**Results:**

Studies on seroprevalence showed 76.62% of goats were seropositive. The total number of animals were divided into different age groups starting from 0-2 months, 2-4 months, 4-6 months, and above 8 months and accordingly highest prevalence of antibodies against ORFV was recorded in the age-group above 8 months of age. Significantly, lower rates of infection were observed in goats of age group 2-4 months. This study recorded that seropositivity from naturally infected animals and in contact apparently healthy animals to be 53.67% and 46.32%, respectively.

**Conclusion:**

The results indicated that CE is a prevalent infection in goats of Assam, and the healthy population is at increased risk of infection.

## Introduction

*Orf virus* (ORFV) is the etiological agent of contagious ecthyma (CE) and is the prototype of the genus *Parapoxvirus* (PPV) which is an epitheliotropic double-stranded DNA genome within the genus PPV, family Poxviridae [1]. The established species in the genus PPV include ORFV, *Pseudocowpox virus*, *Bovine papular stomatitis virus*, and PPV of red deer in New Zealand [2-4]. Members of PPVs, including ORFV, are found to cause infections mostly in domestic and wild ruminants usually affecting young animals with economic and zoonotic implications [5,6]. This virus causes severe exanthematous dermatitis in affected animals characterized by erythematous spots or swelling, followed by formation of papules, nodules, and pustules that progress to develop into thick dry scabs on the skin of lips, oral mucosa, tongue, gingiva, and around the nostrils [7,8]. Although the disease usually resolves in 1-2 months, morbidity of the disease may reach 100% and mortality due to secondary causes may reach 15% [9,10]. There also have been reports of persistent long lasting ORFV infections in goat kids that could last from 3 [11] to 6 months [12]. Persistence of the infection and an increase in its severity are nearly always associated with secondary bacterial infections [13]. Highly vascular nature of the lesions leads to profuse bleeding when injured or biopsied. However, CE infection in sheep and goats is usually self-limiting [14].

As a zoonosis, the virus represents an occupational hazard for farmers, veterinarians, abattoir workers, sheep shearers, and person who prepare animals at home for religious practices [6,14,15]. Transmission of ORFV can be direct and indirectly by contaminated hides and fomites [6]. However, human infection is accidental and first human-to-human transmission has been described by Khan *et al*. [16]. The economic importance of this disease is notable as it is related in causing severe impact in young lambs and kids. The morbidity of the disease can be as high as 100%, but the mortality rate in uncomplicated cases rarely exceeds 1% [12]. The high mortality in young animals is due to the inability of the animals to feed due to oral lesions associated with secondary infections leading to anorexia. Moreover, maggot infestations and secondary bacterial or fungal infections aggravate the condition to worst contributing to mortality [7].

In Assam, a North Eastern part of India, livestock resource is highly livelihood oriented. Animal husbandry plays a significant role in rural economy by providing gainful employment to small and marginal farmers. Goat farming is predominantly practiced across the state as a source of livelihood. In such situations, outbreaks of CE in goat population of Assam cause staid economic loss to farmers as this disease is prevalent in the majority of the areas of the state. It was observed that no systematic control policies are being followed so far against this disease. The present investigation was designed to study the seroprevalence of CE in goats of Assam which will provide an obvious indication about the status of the disease and will help in formulating the control strategy to be applied against this disease.

## Materials and Methods

### Ethical approval

All the procedures have been conducted in accordance with the approval from Institutional Animal Ethics Committee.

### Sample collection

**Figure 1:**
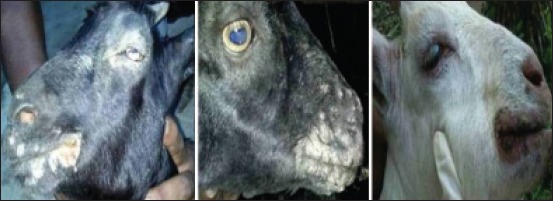
Lesions of contagious ecthyma in goats of Assam.

An investigation of seroprevalence of CE was carried out from a period September 2013 to July 2014. Blood samples were collected from naturally infected (Figure-1) and in contact apparently healthy goats of Assam to screen the prevalence of the disease. Serum was separated and transferred immediately to −20°C freezer for further investigation. A total of 231 serum samples were collected from 12 districts of Assam. The total number of animals was divided into different age groups starting from 0-2 months, 2-4 months, 4-6 months, and above 8 months, and consequently, the prevalence of antibodies against ORFV in different age groups of goats was recorded.

### Maintenance of cells

Primary lamb testes (PLT) cells, P-15 were obtained from Indian Veterinary Research Institute (IVRI), Mukteswar and propagated using Dulbecco’s Modified Eagle’s Medium (DMEM; Sigma-Aldrich) containing 4500 mg glucose/L, 110 mg sodium pyruvate/L and L-glutamine supplemented with 5% fetal calf serum (FCS; HyClone). Antibiotics were added to the growth medium at the rate of penicillin 100 units, streptomycin 100 μg, and amphotericin B 25 μg/ml. For maintaining the cells, maintenance medium containing DMEM with 2% FCS was used.

### Source of reference ORFV

The reference ORFV (Muk 59/05, P-49) was received from IVRI, Mukteswar and kept at 4°C till use.

### Revival and bulk production of reference ORFV

The vaccine seed virus (Orf Muk 59/05, P-49) was revived in PLT cells. Bulk production of the reference virus was carried out in confluent PLT cell culture flasks (300 cc). Lyophilized reference ORFV from passage P-49 was reconstituted in 1 ml of sterile distilled water. The confluent LT monolayer was grown in cell culture flasks (300 cc), and the vaccine seed virus was inoculated onto confluent primary LT monolayer by adding 50 ml of DMEM medium having 2% FCS. The cells were monitored everyday till appearance of cytopathic effects (CPE). The flasks were harvested by repeated freeze-thaw after development of 70-80% CPE. To maintain a uniform virus titer, virus harvests from all the flasks were pooled after the first cycle of freeze-thaw. The harvested virus sample was kept at −80°C until use. The identity of the reference ORFV was further checked by uniplex polymerase chain reaction. Full length of ORFV specific “Major envelope protein (B2L)” was amplified using specific primer sets OVB2LF1, 5´-TCCCTGAAGCCCTATTATTTTTGG-3´and OVB2LR1, 5´GCTTGCGGGCGTT-CGGACCTTC-3 [17] which would yield an expected amplified product of 1206 bp.

### Purification of reference ORFV

ORFV was concentrated and purified using sucrose gradient centrifugation. The harvested sample was subjected to centrifugation at 6000 rpm for 10 min and supernatant was collected into a fresh container. The supernatant was added with PEG 6000 (SRL) at the rate of 8.0% (w/v) and subjected to constant mixing under magnetic stirrer at 4°C overnight. The sample was subjected to centrifugation at 6000 rpm for 30 minutes and the pellet was collected. The pellet was reconstituted in 5 ml of 1× tris acetate ethylene-diamine-tetraacetic acid (EDTA) (TAE) buffer (10 mM) and homogenized and centrifuged again at 7000 rpm for 4 minutes. The sample was overlaid on to 36% sucrose followed by ultracentrifugation at 85,000 ×*g* for 1 h. The resultant pellet was collected and overlaid in between layers of 64% and 36% sucrose gradients and centrifuged at the rate of 80,000 ×*g* for 1 h. The virus present in the translucent layer interfacing the 64% and 36% layers were collected and pelleted after diluting it in TAE buffer. The resultant pellet was collected and resuspended in 1× TAE buffer and stored at −80°C till further use.

### Raising of hyperimmune serum (HIS) against purified ORFV

ORFV specific antibodies were raised in two healthy kids of 8 months to 1 year of age. About 1 ml of purified ORFV antigen, mixed in FCA (Sigma-Aldrich) was divided into two parts (0.5 ml each) and injected intramuscularly at two different sites. One kid was kept as control. The second injection was given by the same route with purified Orf antigen mixed in Freund’s incomplete adjuvant (Sigma-Aldrich) after 14 days of the first injection. Third and fourth injections were given intramuscularly at 10 days interval with 0.5 ml plain antigen. Test bleeding was done after 10 days of the last injection, and the antibody titer was determined by indirect enzyme linked immuno sorbent assay (ELISA) [18,19]. Serum antibody titer showing ≥1280 ELISA titer was aliquoted in 1 ml vials and stored at −20°C until further use.

### Optimization of indirect ELISA

A chequerboard titration was performed for optimization of working dilution of orf antigen and antibodies as per standard protocols. The specific dilution ORFV antigen and standard positive serum that induce approximately 75% absorbance (A492) of the plateau was arbitrarily selected. The reference serum samples from pre-vaccinated and post-vaccinated animals were tested in two-fold dilutions. The antigen and serum dilutions that gave maximal difference in absorbance at 492 nm between positive and negative were selected.

ELISA plates (M/s Nunc, Maxisorp) were coated with purified ORFV with 1:50 dilution (approximately 1 μl/well) in carbonate-bicarbonate buffer (pH 9.6). Antigen was added to all the wells except antigen negative (Ag-ve) control wells, where 100 μl of phosphate buffered saline (PBS) was added. The plates were incubated for 1 h at 37°C and kept overnight at 4°C. After incubation, plates were washed thrice with washing buffer, PBS-T (0.002 mol/L diluted PBS containing 0.05% Tween-20). Blocking buffer (PBS-T with 5% skim milk powder and 3% lactalbumin hydrolysate) at the rate of 100 μl/well was added. The plates were incubated for 1 h at 37°C. Unbound antibodies were washed thrice with washing buffer, PBS-T. Meanwhile two-fold dilutions of serum samples were made in blocking buffer and added at the rate of 50 μl/well and incubated. After incubation, the plate was washed thrice using washing buffer, PBS-T (0.002 mol/L diluted PBS containing 0.05% Tween-20). Diluted serum samples were added to the sample wells. Controls wells included positive and negative sera. The contents were mixed properly by gently tapping the sides of the plate. The plates were again incubated at 37°C for 1 h under constant shaking and washed thrice. A volume of 50 μl diluted anti-goat (1:1000 dilution in blocking buffer) horseradish peroxidase conjugate (A 5420; Sigma-Aldrich) was added to each well and incubated for 1 h at 37°C and washed. 100 μl of freshly constituted substrate solution was added to each well and kept at 37°C without shaking. 4 μl of 3% hydrogen peroxide (H_2_O_2_) per ml of substrate was added and after 15-20 min the reaction was stopped by adding equal volume of 1 M H_2_SO_4_. Optical density (OD) of the wells was measured at 492 nm. Cut-off value was calculated based on negative serum reactivity as follows: (Mean OD value of test sample - Mean OD of negative sample) more than equal ≥0.1 OD was considered as end point of serum dilution.

## Results

### Bulk production and purification of ORFV

**Figure 2:**
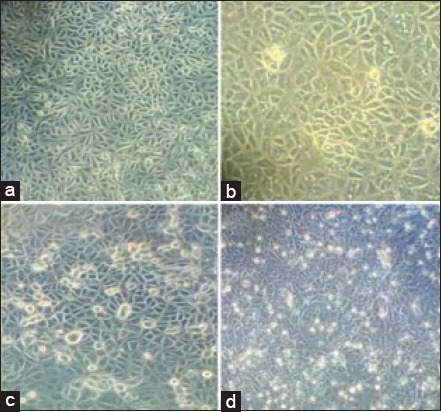
(a) 24 hour confluent PLT cell monolayer, (b) Increased granulation and vacuolation of the monolayer at 2 dpi, (c) Rounding and ballooning of cells at 3-4 dpi, (d) Degeneration of the cell monolayer at 5 dpi.

**Figure 3:**
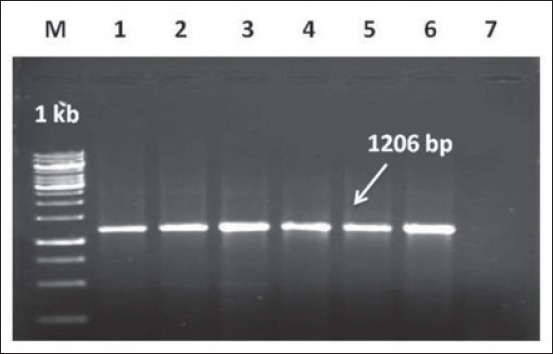
Amplification of full length B2L gene (1206 bp) of reference Orf vaccine virus (Muk59/05), Lane M: DNA Marker 1 kb; Lane 1-6: Orf vaccine virus; Lane 7: NTC.

24 h confluent PLT cell monolayer (Figure-2a) infected with the reference ORFV (Muk 59/05, P-49) in 300 cc flasks showed cytopathic changes. CPE was initiated from the 1^st^ passage itself on day 2 post inoculation (dpi) as increased granulation, rounding and ballooning of cells which finally progressed to 70-80% by day 4-5. Vacuolation of the cytoplasm and degeneration of the cell monolayer was observed at 5-6 dpi (Figure-2b-d). The identity of the reference ORFV was further checked by uniplex PCR of the ‘Major envelope protein B2L’ gene which yielded an amplified product of 1206 bp (Figure-3). A major purified virus band as a well-defined opalescent zone was seen in between 64% and 36% sucrose layers. The virus pellet was finally suspended in required volume of TAE buffer and stored at −80°C till further use.

### Raising of HIS in goats against reference ORFV

Goats immunized with Freund’s complete and incomplete adjuvants mixed with purified ORFV antigen yielded serum antibody titer of 1:1280 and used as tracing antibody in indirect ELISA with a working dilution of 1:100.

### Detection of antibodies against ORFV by indirect ELISA

Out of 231 serum samples collected from 12 districts of Assam, 177 were found seropositive by indirect ELISA with a percent positivity of 76.62%. The highest number of samples having positive antibodies against ORFV was recorded from Lakhimpur district of Assam with a percent prevalence of 92.30% and lowest from Dibrugarh district of Assam with a percent prevalence of 21.42% (Table-1). The total number of animals were divided into different age groups starting from 0-2 months, 2-4 months, 4-6 months, and above 8 months and accordingly the prevalence of antibodies in different age groups of goats were recorded which was found to be highest in the age group above 8 months of age (93/177). Likewise, lowest prevalence was recorded from the age group 2-4 months. The highest number of seropositivity was recorded from naturally infected animals 95/177 (53.67%), while from the in contact apparently healthy animals it was found to be 82/177 (46.32%) (Table-2).

**Table-1:** Detection of antibodies against ORFV in serum samples by indirect ELISA.

Place of collection	Number of serum samples		Total	Number of samples positive	Percent prevalence (%)

	Naturally infected	In contact apparently healthy			
Lakhimpur	20	6	26	24	92.30
Karbi Anglong	4	5	9	8	88.88
Sivasagar	8	12	20	12	60.00
Tinsukia	15	9	24	17	70.83
Nalbari	9	11	20	17	85.00
Nagaon	5	8	13	8	61.53
Dhemaji	8	7	15	13	86.66
Darrang	6	5	11	5	45.45
Dibrugarh	3	11	14	3	21.42
Kamrup	25	17	42	38	90.47
Jorhat	6	8	14	11	78.57
Golaghat	9	14	23	21	91.30
Total	118	113	231	177	76.62

ORFV=*Orf virus*, ELISA=Enzyme linked immunosorbent assay

**Table-2:** Prevalence of antibodies against ORFV in different age groups of goats.

Age groups	Naturally infected animals	In contact apparently healthy animals	Total
2-4 months	9	6	15
4-6 months	15	22	37
6-8 months	18	14	32
Above 8 months	53	40	93
Total	95 (53.67%)	82 (46.32%)	177

ORFV=*Orf virus*

## Discussion

CE is manifested by scabby lesions that commence on the lips, muzzles, mucocutaneous junctions, nostrils and gums of small ruminants, mostly sheep, and goat and can spread to other nonwoolly areas including the legs, feet, and udder [20,21]. The intrinsic immune evasion properties of ORFV coupled with inefficient control measures had probably contributed to the increasing number of outbreaks reported both in sheep and goats [22]. ORFV replicates within the surface layers of the skin as well as the mucosa of the mouth and esophagus [13]. Microabrasions or scarification of the external layer of the skin is necessary before progression of lesions or disease [23,24]. In this study, similar lesions were observed in goats which were confined to the lips and oral commissures. Such abrasions are caused by prickles, thistles, and stubbles as the animals are let loose for grazing which is a common practice among the rural farmers of Assam. Associated with CE, there are other diseases such as *Peste des petits ruminants* and goat pox which need to be differentiated using molecular, serological and histopathological techniques.

In our study, indirect ELISA was optimized to screen the prevalence of CE in the goat population of Assam. Although, in the investigation, all the districts could not be covered, the study covering the areas depict a clear picture of seropositivity of *Orf* antibodies among the goat population. The presence of ORFV specific antibody in sera indicate the occurrence of CE in goats as they were never vaccinated against this virus. A total of 231 blood samples were collected from naturally infected and in contact healthy animals covering 12 districts of Assam, out of which 177 were found seropositive by indirect ELISA with a percent positivity of 76.62%. The difference between the percentage of seropositive and clinically positive goats appeared to be due to the fact that some of the seropositive goats exposed to the virus either recovered or became symptomless carriers. Similar observations were also made in a report by Gokce *et al*. (2005) where they studied about the seroprevalence of CE (Orf) in lambs and humans in the Kars region of Turkey. Considering the age group, the highest prevalence of ORFV-specific antibodies was found in the age group above 8 months of age. A total of 93 goats out of 177 seropositive cases were found to be under the age group above 8 months of age followed by the age group 4-6 (37/177), 6-8 (32/177) and 2-4 months (15/177). The higher seroprevalence of ORFV antibodies in the age group above 8 months might be due to repeated exposure to ORFV infections at different stages of their life. However, collection of more number of samples from goat will be required to elucidate the exact epidemiological picture of CE in Assam.

## Conclusion

The study indicates that CE is endemic in Assam as indicated by high seropositive animals. Human as well as the healthy goat population appears to be at increased risk of infection. Assam being the gateway to the other states of North Eastern Region (NER) of India, this study will provide some baseline data on status of CE among goat population of the entire region. However, it is necessary to conduct detailed seroepidemiological studies throughout the north-eastern region of India and awareness drive among the rural farmers is of great concern so as to prevent severe outbreaks of CE among the healthy goat population of NER in general and Assam in particular.

## Authors’ Contributions

This study was a part of MB’s research work during her M.V.Sc. program. MB carried out the experiment and drafted the final manuscript. DPB and NNB helped in designing the experiments and provided necessary guidelines. SD and BB assisted in sample collection and analysis. All authors have read and approved the final manuscript.
